# Prevalence, virulence genes, and antimicrobial resistance of *Vibrio* species isolated from diseased marine fish in South China

**DOI:** 10.1038/s41598-020-71288-0

**Published:** 2020-08-31

**Authors:** Yiqin Deng, Liwen Xu, Haoxiang Chen, Songlin Liu, Zhixun Guo, Changhong Cheng, Hongling Ma, Juan Feng

**Affiliations:** 1 Key Laboratory of South China Sea Fishery Resources Exploitation and Utilization, Ministry of Agriculture and Rural Affairs, South China Sea Fisheries Research Institute, Chinese Academy of Fishery Sciences, Guangzhou, 510300 China; 2 Tropical Aquaculture Research and Development Centre, South China Sea Fisheries Research Institute, Chinese Academy of Fishery Sciences, Hainan, 572426 China; 3 Key Laboratory of Tropical Marine Bio-Resources and Ecology, South China Sea Institute of Oceanology, Chinese Academy of Sciences, Guangzhou, 510301 China; 4 College of Fisheries and Life Science, Shanghai Ocean University, Shanghai, 201306 China

**Keywords:** Bacteria, Environmental microbiology

## Abstract

Here, 70 potential *Vibrio* pathogens belonging to nine species, dominated by *Vibrio harveyi*, were isolated and identified from diseased aquacultured marine fish in South China. Subsequently, the prevalence of 11 virulence genes and the resistance to 15 antibiotics in these strains were determined. Most strains possessed atypical virulence genes in addition to typical virulence genes. Notably, *hflk* and *chiA* originating from *V. harveyi*, and *flaC* associated with *V. anguillarum* were detected in more than 40% of atypical host strains. Multidrug resistance was widespread: 64.29% strains were resistant to more than three antibiotics, and the multi-antibiotic resistance index ranged from 0.00 to 0.60. The proportions of strains resistant to the antibiotics vancomycin, amoxicillin, midecamycin, and furazolidone all exceeded 50%; nevertheless, all strains were sensitive to florfenicol, norfloxacin, and ciprofloxacin. Furthermore, both virulence genes and antibiotic resistance were more prevalent in Hainan than in Guangdong, owing to the warmer climate and longer annual farming time in Hainan. These results therefore suggest that warming temperatures and overuse of antibiotics are probably enhancing antibiotic resistance and bacterial infection. This study reveals that pathogenic *Vibrio* spp. with multi-antibiotic resistance are highly prevalent among marine fish in South China and thus warrant further attention. The results will provide helpful guidance for ecological regulation and local antibiotic use in the control of marine fish farming’ *Vibrio* diseases in South China, facilitating the implementation of national green and healthful aquaculture.

## Introduction

To meet the increasing demand for animal protein, the aquaculture industry has developed rapidly, and the proportion of fish farming (marine fish farming and freshwater fish farming) increased from 5% to more than 40% of global fish production since 1970^1^. Notably, in China, fish farming production accounted for as much as 75% of the total fish production in 2018^2^. Because of the advantage of the proximity to the South China Sea, farming of marine fish (including *Lateolabrax japonicus*, *Panalichthys lethostigma*, *Pseudosciaena crocea, Rachycentron canadum*, and *Epinephelussp* spp*.*) has rapidly developed in South China^2,3^, accounting for 78.26% of the total marine fish farming production in China in 2018^2^. In recent years, intensive and industrialized mariculture has gradually developed in South China to meet the needs of the national economy and provide food support^3^. Therefore, sustainable development of marine fish farming in South China is crucial to securing the food supply and strengthening the national economy of China.

However, over-intensive aquaculture and harmful anthropogenic activities contribute to outbreaks of serious bacterial infections, thereby affecting both economic and social development^4,5^. Vibriosis is one of the most prevalent bacterial diseases affecting diverse marine fish and shellfish^6^. Chong et al.^7^ have noted that approximately two-thirds of the diseases reported in *Epinephelus* spp. are vibrioses, which affect all stages of fish growth and lead to as much as 50% mortality among fish^8,9^. Several species of Vibrionaceae, including *Vibrio harveyi, V. vulnificus*, *V. parahaemolyticus*, *V. alginolyticus*, and *V. anguillarum*, are the most common species and are associated with health problems in marine animals^10^. For example, infection with *V. alginolyticus* and *V. harveyi* in marine cage-cultured *P. crocea* led to a mortality rate between 30 and 40%, and as high as 80% in Zhejiang province, China, between May 2000 and November 2003^11^.

The pathogenicity of *Vibrio* strains is facilitated by a broad range of virulence factors encoded by virulence genes^12^. In general, virulence factors allow pathogens to infect and damage the host, by enabling pathogenic adherence and entrance, establishment and multiplication, avoidance of host defenses, damage to the host, and finally exit from the infected host^13^. Five major virulence factors are found in vibrios: capsular polysaccharides, adhesive factors, cytotoxins, lipopolysaccharides, and flagella^14,15^. Bacteria acquire new (atypical) virulence genes in addition to their innate virulence genes (typical virulence genes), thus improving their virulence. These atypical virulence genes are acquired via horizontal gene transfer (HGT)^12^ from the environment and/or other bacteria^16^. HGT, particularly the HGT of atypical virulence genes, is considered an important process influencing bacterial evolution and promoting bacterial virulence^17^. Global climate change, antibiotics, heavy metals, and nutrient pollutants have been reported to increase the pathogenicity and drug resistance of pathogens by affecting HGT^18,19^. Therefore, the investigation of virulence genes, particularly atypical virulence genes, and the analysis of factors influencing the presence of virulence genes should provide meaningful insights for the study of *Vibrio* pathogenesis and facilitate the establishment of ecological control systems.

Antibiotics have been extensively used against bacterial infections in the aquaculture industry^20^. For instance, florfenicol and oxolinic acid are mainly used to control vibriosis in cod fry in Norway^21^. Quinolones and flumequine are widely used to treat classical and cold water vibriosis^22^. However, the extensive use of antibiotics results in drug residues, thus promoting the development of antibiotic resistance and consequently contaminating food, water, and sediments^23^. Defoirdt et al.^24^ have reported that with the increased use of antibiotics such as chloramphenicol, cotrimoxazole, erythromycin, and streptomycin to combat *V. harveyi* infection in giant tiger prawns, the bacteria have become resistant, and the antibiotics are no longer effective. In 2013, 54,000 tons of antibiotics were excreted into the environment by humans and animals in China, and this amount has gradually increased in subsequent years^25^, resulting in the selection and accumulation of severely resistant and multi-antibiotic resistant bacteria^26^. Moreover, strong drug resistance is likely to enhance bacterial virulence, making it difficult to treat infections^27,28^. Therefore, analyzing the drug resistance of pathogens in a specific area is crucial to formulating local antibiotic reduction policies and effective antibiotic use programs.

High-density farming, along with intense human activities and global climate change, has led to frequent vibriosis, antibiotic overuse, and antibiotic resistance in recent years^6,29^. To thoroughly deploy the "Green and Healthy Aquaculture" initiative launched in 2020, the Ministry of Agriculture and Rural Affairs decided to implement “Drug Reduction in Aquaculture” and “Ecological Health Breeding” initiatives for the development of aquaculture animal disease detection, pathogen resistance monitoring, determination of suitable local antimicrobial profiles for aquaculture, provision of guidance for scientific medication, and promotion of vaccine use for disease prevention. Previous studies have reported the virulence genes and antibiotic resistance of *V. harveyi* in South China^30,31^; however, little information on other *Vibrio* spp. is available regarding the virulence genes and antimicrobial resistance patterns in South China. In this study, we assessed the variations in nine *Vibrio* species’ virulence genes and antibiotic resistance, and identified the factors likely to influence the transmission of virulence and drug resistance. Our results may provide guidance for ecological regulation and local antibiotic use for sustainable and healthful breeding, facilitating the implementation of national green and healthful aquaculture.

## Results

### Isolation of *Vibrio* species from diseased marine fishes

**Figure 1:**
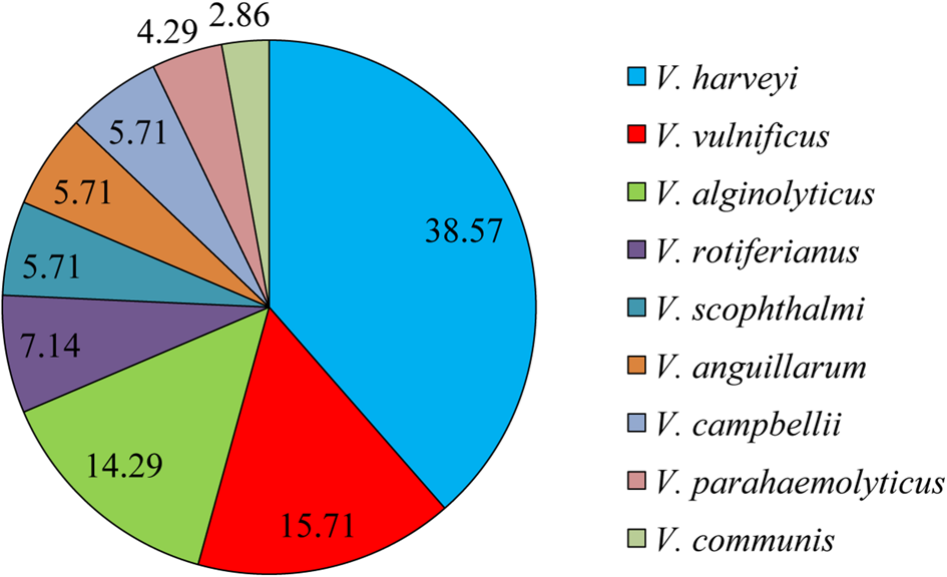
The *Vibrio* species composition (%).

A total of 70 *Vibrio* species were collected from diseased marine fishes in an aquaculture area in South China. These bacterial species accounted for approximately 70% of the dominant clones those were collected from 2216E, nutrient, and Brain–Heart Infusion (BHI) agar plates. Among them, 27 *V. harveyi*, 11 *V. vulnificus*, 10 *V. alginolyticus*, 5 *V. rotiferianus*, 4 *V. scophthalmi*, 4 *V. anguillarum*, 4 *V. campbellii*, 3 *V. parahaemolyticus*, and 2 *V. communis* were identified, accounting for 38.57%, 15.71%, 14.29%, 7.14%, 5.71%, 5.71%, 5.71%, 4.29%, and 2.86% of the total strains, respectively (Fig. 1). The phylogenetic tree showed that each strain clustered with the corresponding reference strain and was separate from other reference strains (Figure S1). Among the strains, 32 and 14 were isolated from Guangdong and Hainan, respectively (Table S1, Table S2).

### Distribution of virulence genes in *Vibrio* isolates

Eleven virulence genes were detected with the multi-virulence gene indexes (MVGIs = the number of virulence genes detected in one strain/total number of detected virulence genes) from 0.00 to 0.82 (Table S1). Generally, six *V. harveyi* typical virulence genes (*ahpA*, *vhh*, *hflK*, *luxR*, *chiA*, and *toxR*_*Vh*_) were widely present in *V. harveyi* isolates, with positivity rates of 70.37%, 70.37%, 44.44%, 81.48%, 88.89%, and 88.89%, respectively (Table 1). The *V. vulnificus* hemolysin gene *vvh* was detected in 18.18% (2/11) of *V. vulnificus* isolates (Table 1). The *V. parahaemolyticus* thermostable direct hemolysin gene *tdh* was present in 33.33% (1/3) of *V. parahaemolyticus* isolates (Table 1). The *V. anguillarum* flagella C subunit gene *flaC* was present in all four *V. anguillarum* strains (Table 1). Specially, all nine virulence genes and two *V. cholera* specific genes, *toxRVc* and *hlyA*, were detected in their corresponding atypical hosts, with positivity rates of 25.58%, 27.91%, 41.86%, 18.60%, 51.16%, 23.26%, 28.81%, 11.94%, 56.06%, 17.14%, and 12.86%, respectively (Table 1).

**Table 1:** The presence of virulence genes.

Hosts	Virulence genes	Total rate (%)	The rate in typical hosts (%)	The rate in atypical hosts (%)
*V. harveyi*	*ahpA*	42.86	70.37	25.58
	*vhh*	44.29	70.37	27.91
	*hflk*	42.86	44.44	41.86
	*luxR*	42.86	81.48	18.60
	*chiA*	65.71	88.89	51.16
	*toxRVh*	48.57	88.89	23.26
*V. vulnificus*	*vvh*	27.14	18.18	28.81
*V. parahaemolyticus*	*tdh*	12.86	33.33	11.94
*V. anguillarum*	*flaC*	58.57	100.00	56.06
*V. cholerae*	*toxRVc*	17.14	–	17.14
	*hlyA*	12.56	–	12.86

### Antimicrobial resistance profiles of *Vibrio* isolates

**Figure 2:**
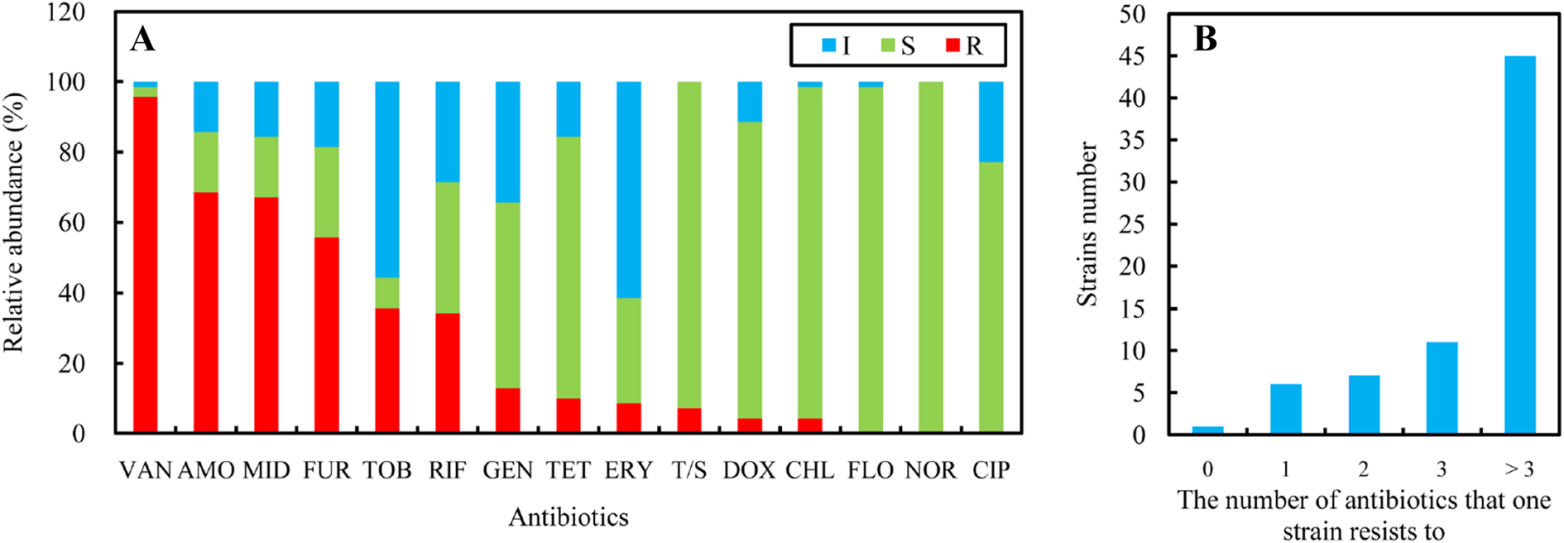
Antibiotic resistance of *Vibrio* isolates. (**A**) The relative abundance of intermediate (I), resistant (R), and sensitive (S) strains. (**B**) The number of strains resistant to 0, 1, 2, 3, and more than 3 antibiotics. VAN: vancomycin, AMO: amoxicillin, MID: midecamycin, FUR: furazolidone, TOB: tobramycin, RIF: rifampicin, GEN: gentamicin, TET: tetracycline, ERY: erythromycin, T/S: trimethoprim-sulfamethoxazole, DOX: doxycycline, CHL: chloramphenicol, FLO: florfenicol, NOR: norfloxacin, and CIP: ciprofloxacin.

The antimicrobial resistance profiles of the *Vibrio* species are illustrated in Fig. 2A,B, Figure S2, and Table S2. The results showed high resistance (> 50%) to vancomycin (95.71%), amoxicillin (68.57%), midecamycin (67.14%), and furazolidone (55.71%); moderate resistance (10%–50%) to tobramycin (35.71%), rifampicin (34.29%), gentamicin (12.86%), and tetracycline (10.00%); and low resistance (< 10%) to erythromycin (8.57%), trimethoprim-sulfamethoxazole (7.14%), doxycycline (4.29%), and chloramphenicol (4.29%) (Fig. 2A). Sensitivity was observed for florfenicol (0.00%), norfloxacin (0.00%), and ciprofloxacin (0.00%) (Fig. 2A). The multi-antibiotic resistance indexes (MARIs = the number of antibiotics with resistance detected in one strain/total number of detected antibiotics) ranged from 0.00 to 0.60 (Table S2). In total, 38 resistance types (A to AL) were present among all strains, and the antibiotic resistance pattern abundance (ARPA = the number of resistance types/the number of strains) was 0.54. The ang_X14RP15 showed sensitivity to all 15 tested antibiotics. Six strains had resistance to one tested antibiotic, with resistance types of AD, AH, and AI, of which four were resistant to vancomycin, one was resistant to midecamycin, and one was resistant to furazolidone (Fig. 2B, Figure S2). Seven strains showed resistance to two tested antibiotics (AE, AG, and AK), all of which were resistant to vancomycin (Fig. 2B, Figure S2). In addition, three strains showed resistance to amoxicillin, one showed resistance to midecamycin, and three showed resistance to furazolidone (Fig. 2B, Figure S2). Eleven strains had resistance to three tested antibiotics (C, D, AB, AF, AJ, and AL), all of which were resistant to vancomycin and two of which were resistant amoxicillin, midecamycin, furazolidone, tobramycin, and gentamicin (Fig. 2B, Figure S2). The other 45 (64.29%) strains had multidrug resistance to more than three tested antibiotics (Fig. 2B, Figure S2). The alg_F14DM01 (AA) exhibited the highest MARI value of 0.60 and showed resistance to nine antibiotics (Figure S2).

### Spatial variations in virulence genes and antimicrobial resistance

**Figure 3:**
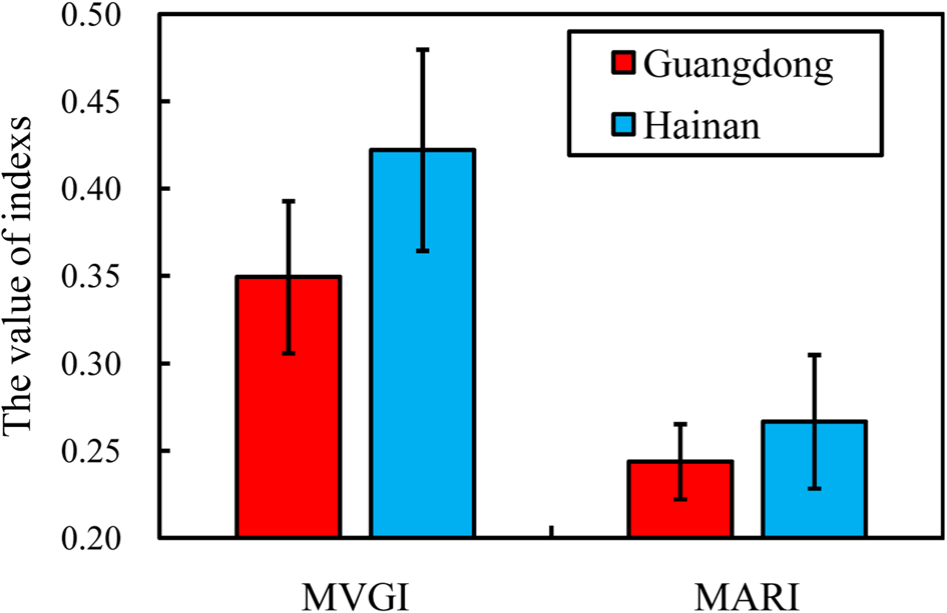
The multi-virulence gene indexes (MVAI) and multi-antibiotic resistance indexes (MARI) in Guangdong and Hainan.

A total of 14 resistance types were found in the strains collected from Guangdong, with an ARPA of 0.44, whereas ten resistance types were found in the strains collected from Hainan, with an ARPA of 0.71 (Figure S2). Although the MVGIs and MARIs were not significantly different between Guangdong and Hainan (*t*-test, all *p* > 0.05), the average MVGI and MARI were higher in Hainan (0.42 and 0.27, respectively) than in Guangdong (0.35 and 0.24, respectively) (Fig. 3). Specifically, higher prevalence of *aphA*, *vhh*, *hflK*, *chiA*, and *flaC* and higher resistance to vancomycin, amoxicillin, furazolidone, tobramycin, gentamicin, and doxycycline were found in Hainan (virulence-associated genes rates of 64.29%, 57.14%, 64.29%, 78.57%, and 64.29%, respectively, and antimicrobial resistance rates of 100.00%, 57.14%, 57.14%, 42.86%, 28.57%, and 7.14%, respectively) than in Guangdong (virulence-associated gene prevalence of 37.50%, 37.50%, 40.63%, 53.12%, and 50.00%, respectively, and antimicrobial resistance rates of 90.63%, 50.00%, 34.38%, 40.63%, 9.38%, and 6.25%, respectively) (Figure S3A,B).

## Discussion

The presence of pathogenic *Vibrio* spp. in marine fishes is gaining attention, because these organisms frequently cause of systemic infections resulting in fish death and even diseases in humans^32^. Here, we collected the dominant clones from diseased marine fishes. Nine *Vibrio* species that are potential pathogens in fish diseases were isolated. *V. harveyi* was most frequently isolated, followed by *V. vulnificus* and *V. alginolyticus*. Among them, *V. harveyi*, *V. alginolyticus*, *V. rotiferianus*, *V. campbellii*, *V. parahaemolyticus*, and *V. communis* (a total of 51 isolates, accounting for 72.86%) belonged to *Harveyi* clade strains, the most important pathogen clade in aquatic organisms^13^. *Harveyi* clade species prefer warm temperatures and are expected to be transmitted to higher latitudes with global climate change^33,34^. Vibriosis has been frequently recorded in tropical Malaysia, which has a year-round tropical climate of 28 °C, and *V. harveyi* is most frequently isolated in outbreaks, followed by *V. parahaemolyticus*, *V. alginolyticus*, and *V. anguillarum*, which affect perches and groupers^32,35^. Therefore, the warming temperatures in semitropical/tropical South China should contribute to the extensive isolation of the *Harveyi* clade, particularly *V. harveyi*, from diseased marine fishes. *V. vulnificus* and *V. parahaemolyticus* are also pathogenic to humans, causing serious diseases including seafood-borne gastroenteritis, wound infections, and septicemia^6^. The *Harveyi* clade, particularly *V. harveyi*, is prevalent in marine fish farming in South China and has led to dramatic losses in the aquaculture industry.

Identification of virulence factors is essential for evaluating bacterial pathogenicity, because these factors allow bacteria to infect and damage hosts^32^. In this study, the high prevalence (> 70%) of hemolysin *vhh*, the quorum-sensing regulator *luxR*, chitinase *chiA*, and the transmembrane transcription regulator *toxR*_*Vh*_ in the typical host *V. harveyi* was consistent with findings from previous reports, whereas the moderate presence of serine protease *hflk* (44.44%) was lower than that previously reported^30,31^. Except for the *V. anguillarum* flagella gene *flaC* identified in all *V. anguillarum* strains, *V. parahaemolyticus* hemolysin *tdh* and *V. vulnificus* hemolysin *vhh* were observed in less than one-third strains of *V. parahaemolyticus* and *V. vulnificus*, respectively. Previous studies have indicated that virulence genes can be inherent (typical virulence genes) and/or obtained by HGT (atypical virulence genes)^12^. The nine above-mentioned genes and two virulence genes specific to *V. cholera* (*toxR*_*Vc*_ and *hlyA*) were detected in their atypical hosts; notably, *hflK*, *chiA*, and *flaC* were found in more than 40% of atypical host strains, findings similar to those from studies on strains isolated from regions including Mexico, USA, Thailand, Japan, and Spain^13^. We speculate that HGT plays an important role in the evolution and virulence development in *Vibrio* spp. Moreover, these virulence factors may be essential for virulence toward different hosts, because vibrios infect a wide range of aquatic hosts, including fish, shrimp, and mollusks^6,32,36^. The acquisition of atypical virulence genes may increase *Vibrio* virulence against a specific host, although establishing an obvious correlation between the pathogenicity and the number/kind of virulence genes in vibrios is difficult.

Antibiotics are widely used to prevent or treat bacterial diseases in aquaculture, resulting in an increase in antibiotic resistance and multidrug resistance in bacteria and making treatment of infections difficult^28^. In the present study, the ARPA of 0.54, the highest MARI of 0.60, and the multidrug resistance rate of 64.29% were all higher than findings from previous studies in the same area^37,38^, indicating that antimicrobial resistance is temporally different and is likely to increase with time. In this study area, compared with other areas, we found that resistance to vancomycin, amoxicillin, and furazolidone was most prevalent (> 50%), resistance to tetracycline was less prevalent (10%–50%), and sensitivity (< 10%) to chloramphenicol and norfloxacin was observed, results consistent with findings from studies in South India and the Persian Gulf^39–41^. The low resistance to gentamicin and erythromycin were similar to findings from studies in South India and Persian Gulf, respectively^39–41^. The moderate resistance to tobramycin was higher than that in South India^40^, whereas the low resistance to doxycycline was much lower than that in the Persian Gulf ^41^. These results indicated that antimicrobial resistance shows spatial variation, probably because of the types of antibiotics used and the development of different antibiotic resistance mechanisms^42,43^. For example, extensive intrinsic resistance to vancomycin and amoxicillin has been reported^44,45^. Use of antibiotics for which high or moderate resistance have been observed (including vancomycin, amoxicillin, midecamycin, furazolidone, tobramycin, rifampicin, gentamicin, and tetracycline) is recommend to be reduced, whereas antibiotics for which low resistance and sensitivity has been reported (including erythromycin, trimethoprim-sulfamethoxazole, doxycycline, chloramphenicol, florfenicol, norfloxacin, and ciprofloxacin) are suggested to be used in marine fish farming to inhibit bacterial diseases. However, chloramphenicol and norfloxacin have been banned in the aquaculture industry by the Ministry of Agriculture and Rural Affairs, China.

In Hainan, compared with Guangdong, both virulence genes and antibiotic resistance were more prevalent, probably because of the warmer temperature and more useage of antibiotics in Hainan. Warming temperatures can directly induce the expression of antibiotic resistance genes and virulence genes^45^. For instance, the virulence factors involved in motility, host degradation, secretion, antimicrobial resistance, and transcriptional regulation are upregulated as much as 16 times in *V. corallilyticus* when the temperature is increased from 24 to 27 °C, concurrently with phenotypic changes in motility, antibiotic resistance, hemolysis, cytotoxicity, and bioluminescenc^46^. Temperature has also been reported to affect HGT, in an important mechanism of virulence and drug resistance transmission, through influencing functions including biofilm formation, membrane permeability, immune system activity, and HGT-related enzymes^47^. The conjugation efficiency of *Pseudomonas* spp. has been found to increase by 10,000 times after a temperature increase from 15 °C to 28 °C by promoting the biofilm formation of *Pseudomonas* spp^48^. Moreover, extensive vibriosis outbreaks in summer have been widely reported^32,49^. As the southernmost province in China, Hainan is located in a lower latitude (18°10′–20°10′ N, 108°37′–111°03′ E) than that of Guangdong (20°13′–25°31′ N, 109°39′–117°19′ E), and it consequently is usually approximately 3 °C warmer than Guangdong (Figure S4A). Because of warming temperatures, Hainan became a major marine fish hatchery and usually has a longer annual breeding time than Guangdong, resulting in more usage of antibiotics (Figure S4B, Table S3) and enhanced antibiotic resistance in Hainan, which has also been supported by the temporally increased antimicrobial resistance in South China^37^. Additionally, acid–base and organic pollution have also been reported to promote HGT and enhance bacterial virulence and antibiotic resistance^50^.

In conclusion, vibrios, particularly the *Harveyi* clade, cause serious diseases in marine fish, threatening the sustainable development of the aquaculture industry in South China. Virulence genes and antimicrobial resistance are prevalent in those isolated *Vibrio* strains. Notably, atypical virulence genes are presented in *Vibrio* strains, promoting bacterial virulence and broadening the host range. High multi-drug resistance makes it difficult to treat infections. Warm temperatures and continued use of antibiotics are likely to enhance the bacterial virulence and antimicrobial resistance. Our results may be helpful for evaluating *Vibrio* pathogenicity and the abuse of antibiotics. We expect that they can provide guidance for future disease treatment in aquaculture, and serve as a theoretical basis for the development of sustainable disease control methods. To prevent and control the diseases caused by *Vibrio* species in South China, we suggest the following. (1) In aquaculture, particularly in indoor culture, ecological control systems, including temperature control, should be established, and the use of antibiotics, particularly vancomycin, amoxicillin, midecamycin, furazolidone, tobramycin, rifampicin, gentamicin, and tetracycline, should be reduced to minimize the expression of virulence and resistance genes and decrease the transmission of virulence and resistance. Our results may be useful to support the actions of “Drug Reduction in Aquaculture” and “Ecological Health Breeding.” (2) In the absence of other eco-friendly control methods, erythromycin, trimethoprim-sulfamethoxazole, doxycycline, florfenicol, and ciprofloxacin should be used to inhibit *Vibrio* diseases. Our findings highlight the need for more detailed investigations to support the control of antibiotic use, formulation of relevant laws, and improvements in consumer protection and public health safety. Future research should focus on the following: (1) the detailed virulence mechanism, with the aim of developing environmentally friendly treatments, such as vaccines, probiotics, and immunostimulants; and (2) the joint influence of environmental and anthropogenic factors on the virulence and antibiotic resistance of *Vibrio* populations in large areas over long time scales, to aid in establishing ecological regulations in the aquaculture industry.

## Methods

### Bacterial isolates

The diseased marine fishes (including *Protonibea* spp., *Epinephelussp* spp., *Siganus* spp., *Trachinotus* spp., and *Nibea* spp., etc.) were determined by clinical diagnosis, with showing a frail situation of sluggish action and reducing ingestion and having the typical symptoms of nodule, rotten body, ascites, ulcer, or enteritis etc. Then, the *Vibrio* species were isolated and identified from the diseased marine fishes collecting in Guangdong province (including the cities of Zhuhai, Zhanjiang, Huizhou, Chaozhou, and Shenzhen) and Hainan province (including Xincun bay and the cities of Sanya and Wenchang) with following procedures. The tissues (including the liver, spleen, kidney, intestines, and brain) of diseased marine fishes were homogenized. Then the homogenates were screened on 2216E, nutrient, and Brain–Heart Infusion (BHI) agar plates simultaneously and incubated at 28 °C for 24–48 h. The dominant clones were selected and twice purified on the same agar plates. Then, one signal clone was inoculated with fresh 2216E, nutrient, or BHI broth. The genomic DNA was extracted with a Bacterial genomic DNA Extraction Kit (Tiangen, China) and quantified with 0.75% agarose gel electrophoresis and NanoDrop 2000 spectrophotometry (Thermo Fisher Scientific, USA). With the genomic DNA as a template, the 16S rRNA genes of the clones were amplified and sequenced with the universal primers 8F/1492R. Subsequently, *Vibrio* spp. strains were chosen for species identification on the basis of the *rctB* gene with the primers *rctB*-F/R. A phylogenetic tree was constructed with the *rctB* gene sequences of the isolates and 27 reference strains from the National Center of Biotechnology Information (NCBI) database by using the Kimura 2-parameter model and the neighbor-joining method, with bootstrapping 1,000 times via MEGA6.0 software. The primer sequences used for this study are shown in Table S4.

### Detection of virulence genes

To evaluate the distribution of virulence genes in *Vibrio* species, we analyzed six typical *V. harveyi* virulence genes (*ahpA*, *vhh*, *hflK*, *luxR*, *chiA*, and *toxR*_*Vh*_), the *V. vulnificus* hemolysin gene *vvh*, the *V. parahaemolyticus* thermostable direct hemolysin gene *tdh*, the *V. anguillarum* flagella C subunit gene *flaC*, and two *V. cholera* typical virulence genes (*toxRVc* and *hlyA*) with PCR. The PCR was performed in 20 μL reactions containing 10.0 μL of Premix Taq (TaKaRa Taq Version 2.0 plus dye) (Takara, Japan), 1.0 μL of each primer (10 μM), 1.0 μL of template DNA (20 ng/L), and 7.0 μL of sterilized water. The amplification was performed in an automatic thermal cycler (Bio-Rad, USA) as follows: initial denaturation at 95 °C for 5 min; 35 cycles of denaturation at 95 °C for 30 s, annealing at the annealing temperature for 30 s, and extension at 72 °C for 60 s/kb; and final extension at 72 °C for 10 min. The PCR amplification products were assessed with 1.0% agarose gel electrophoresis.

### Antimicrobial resistance assays

Antimicrobial resistance assays were performed with the disk diffusion method according to Clinical and Laboratory Standards Institute (CLSI) guidelines (Wayne, 2011). A total of 15 antibiotics were assessed: furazolidone (FUR, 300 μg/disk), erythromycin (ERY, 150 μg/disk), gentamicin (GEN, 10 μg/disk), rifampicin (RIF, 5 μg/disk), norfloxacin (NOR, 10 μg/disk), ciprofloxacin (CIP, 50 μg/disk), chloramphenicol (CHL, 30 μg/disk), florfenicol (FLO, 30 μg/disk), tetracycline (TET, 30 μg/disk), trimethoprim-sulfamethoxazole (T/S, 23.75/1.25 μg/disk), amoxicillin (AMO, 20 μg/disk), vancomycin (VAN, 30 μg/disk), tobramycin (TOB, 10 μg/disk), midecamycin (MID, 30 μg/disk), and doxycycline (DOX, 300 μg/disk). Briefly, Mueller–Hinton agar plates with 2% sodium chloride were swabbed with overnight bacterial cultures. Different antibiotic discs with 6 mm diameters were placed onto the plates and incubated at 28 °C for 24 h. The diameter of the inhibition zone was measured, and the results were interpreted as sensitive, intermediate, or resistant on the basis of the manufacturers’ and CLSI M45-A guidelines^51^. *Escherichia coli* ATCC 35,218 was used as a quality control strain in each run.

### Statistical analysis

The MVGIs and MARIs were compared between Guangdong and Hainan by using Student’s *t*-test, and *p* < 0.05 was considered to indicate a significant difference.

### Ethics statement

All fish handling and experimental procedures were approved by the Animal Care and Use Committee of South China Sea Fisheries Research Institute, Chinese Academy of Fishery Sciences. All experiments were performed in accordance with the relevant guidelines and regulations.

## Supplementary information

Supplementary information.
