# Supplementary material for: Prevalence, virulence genes, and antimicrobial resistance of Vibrio species isolated from diseased marine fish in South China
Source: Sci Rep. 2020 Aug 31;10:14329. doi: 10.1038/s41598-020-71288-0 (PMC7459350; doi:10.1038/s41598-020-71288-0)
Supplement: Supplementary file 1 — Supplementary information. [file 41598_2020_71288_MOESM1_ESM.doc]

**Scientific Reports**

**Prevalence, virulence genes and antimicrobial resistance of *Vibrio* species isolated from diseased marine fish in South China**

Yiqin Deng^1, 2^, Liwen Xu^1^, Haoxiang Chen^1, 4^, Songlin Liu^3^, Zhixun Guo^1^, Changhong Cheng^1^, Hongling Ma^1^, Juan Feng^1, 2*^

^1^ Key Laboratory of South China Sea Fishery Resources Exploitation & Utilization, Ministry of Agriculture and Rural Affairs, South China Sea Fisheries Research Institute, Chinese Academy of Fishery Sciences, Guangzhou 510300, China

^2^ Tropical Aquaculture Research and Development Centre, South China Sea Fisheries Research Institute, Chinese Academy of Fishery Sciences, Hainan, 572426, China

^3^ Key Laboratory of Tropical Marine Bio-resources and Ecology, South China Sea Institute of Oceanology, Chinese Academy of Sciences, Guangzhou 510301, China

^4^ College of Fisheries and Life Science, Shanghai Ocean University, Shanghai 201306, China

*Correspondence:

**Juan Feng:** South China Sea Fisheries Research Institute, Chinese Academy of Fishery Sciences, Guangzhou 510300, China, Email: [juanfeng@scsfri.ac.cn](mailto:juanfeng@scsfri.ac.cn), Tel: +86-020-89108320

**Table S1.** The presence of virulence genes in *Vibrio* strains

| Strain name | *V. harveyi* | | | | | | *V. vulnificus* | *V. parahaemolyticus* | *V. anguillarum* | *V. cholerae* | |  | Province |
| --- | --- | --- | --- | --- | --- | --- | --- | --- | --- | --- | --- | --- | --- |
|  | *ahpA* | *vhh* | *hflk* | *luxR* | *chiA* | *toxR* | *vvh* | *tdh* | *flaC* | *toxR_VC_* | *hlyA* |  |  |
| har_11QS22 | + | + | + | + | + | + | + | - | + | - | + | 0.82 | Guangdong |
| har_11WS31 | - | - | - | - | + | - | - | - | - | + | - | 0.18 | Guangdong |
| har_11WS38ABC | - | - | - | - | + | - | - | - | - | - | - | 0.09 | Guangdong |
| har_14K | - | - | - | - | - | + | - | + | + | - | - | 0.27 | NK |
| har_BSE1 | - | - | + | - | - | + | - | - | - | - | - | 0.18 | NK |
| har_BSE4 | + | + | + | + | + | + | - | - | + | - | - | 0.64 | NK |
| har_D79 | + | + | - | + | + | + | - | + | + | - | - | 0.64 | NK |
| har_F14MM01 | + | + | - | + | + | + | - | + | + | - | - | 0.64 | NK |
| har_F14MM02 | + | + | - | + | + | + | - | + | + | - | - | 0.64 | NK |
| har_FE1 | + | + | - | + | + | + | + | - | + | - | + | 0.73 | NK |
| har_FE2 | - | - | - | + | + | + | - | - | + | - | + | 0.45 | NK |
| har_JT1 | + | + | - | + | + | + | - | - | + | - | - | 0.55 | NK |
| har_JT2 | + | + | - | + | + | + | - | - | + | - | + | 0.64 | NK |
| har_JT3 | - | + | + | + | + | + | - | - | + | - | + | 0.64 | NK |
| har_V12RP10 | - | - | - | + | + | - | - | + | + | - | + | 0.45 | Guangdong |
| har_V12XC32 | - | - | - | - | - | + | - | - | + | + | - | 0.27 | Hainan |
| har_V13ZH01 | + | + | - | + | + | + | - | + | - | - | - | 0.55 | Guangdong |
| har_X11HK26 | + | + | - | + | + | + | - | - | + | - | + | 0.64 | Guangdong |
| har_X12RP11 | + | + | + | + | + | + | - | - | - | - | - | 0.55 | Guangdong |
| har_X12XW01 | + | + | + | + | + | + | - | - | + | - | - | 0.64 | Guangdong |
| har_X13DS03 | + | + | + | + | + | + | - | + | - | - | - | 0.64 | Fujian |
| har_X13RP16 | + | + | + | + | + | + | - | - | - | - | - | 0.55 | Guangdong |
| har_X13SY09 | + | + | + | + | + | + | - | - | + | - | - | 0.64 | Hainan |
| har_X13ZH05 | + | + | + | + | + | + | - | - | + | - | - | 0.64 | Guangdong |
| har_X14XC01 | + | + | + | + | + | + | - | - | - | - | - | 0.55 | Hainan |
| har_X14XC17 | + | - | + | + | + | + | - | - | + | - | - | 0.55 | Hainan |
| har_X15SZ09 | + | + | - | + | + | + | + | - | + | - | - | 0.64 | Guangdong |
| vul_X13ZH02 | - | - | - | - | - | - | - | - | - | - | - | 0.00 | Guangdong |
| vul_X13ZH04 | - | - | - | - | - | - | - | - | - | - | - | 0.00 | Guangdong |
| vul_X13ZJ03 | - | - | - | - | - | - | - | - | - | + | - | 0.09 | Guangdong |
| vul_X14DS02 | - | - | - | - | + | - | - | - | - | - | - | 0.09 | Fujian |
| vul_X14HD02 | - | - | - | - | - | - | - | - | - | + | - | 0.09 | Guangdong |
| vul_X14RP3101 | - | + | + | + | + | - | + | + | - | - | - | 0.55 | Guangdong |
| vul_X14SZ02 | - | - | - | - | - | - | - | - | - | - | - | 0.00 | Guangdong |
| vul_X14SZ04 | - | - | - | - | - | - | - | - | - | - | - | 0.00 | Guangdong |
| vul_X14SZ13 | - | - | - | - | + | + | - | - | - | - | - | 0.18 | Guangdong |
| vul_X14XC21 | - | - | - | - | - | - | - | - | - | - | - | 0.00 | Hainan |
| vul_X16XC06 | - | - | - | - | - | - | + | - | - | - | - | 0.09 | Hainan |
| alg_11QS15 | - | + | - | - | + | - | + | - | + | - | - | 0.36 | Guangdong |
| alg_E09811 | - | - | - | - | + | - | - | - | + | - | - | 0.18 | NK |
| alg_E09823 | - | - | + | - | - | - | - | - | - | - | - | 0.09 | NK |
| alg_F1 | - | - | + | - | + | - | - | - | + | + | - | 0.36 | NK |
| alg_F14DM01 | - | - | - | - | - | - | + | - | - | - | - | 0.09 | NK |
| alg_L12LF0301 | - | - | + | - | + | - | - | - | - | + | - | 0.27 | NK |
| alg_L12LF0302 | - | - | - | - | - | - | + | - | - | - | - | 0.09 | NK |
| alg_L12LF07 | - | - | + | - | + | - | + | - | + | + | - | 0.45 | NK |
| alg_N14FS02 | - | - | - | - | - | + | + | - | - | + | - | 0.27 | NK |
| alg_V12YD1601 | - | - | + | - | + | - | - | - | - | - | - | 0.18 | Hainan |
| rot_JT1 | - | + | - | - | + | - | - | - | + | - | - | 0.27 | NK |
| rot_X12XC39 | - | - | - | - | + | - | + | - | - | - | + | 0.27 | Hainan |
| rot_X13SY03 | + | + | + | - | + | + | + | - | + | - | - | 0.64 | Hainan |
| rot_ZHG1 | - | - | - | - | + | - | - | - | + | - | - | 0.18 | NK |
| rot_ZHG2 | + | + | - | - | + | - | - | - | + | - | - | 0.36 | NK |
| sco_X14RP05 | - | - | + | - | - | - | - | - | - | - | - | 0.09 | Guangdong |
| sco_X14RP08 | + | - | + | - | - | + | + | - | + | + | - | 0.55 | Guangdong |
| sco_X14RP0801 | - | - | + | - | - | + | - | - | + | + | - | 0.36 | Guangdong |
| sco_X14RP09 | - | + | + | + | - | + | - | - | + | - | - | 0.45 | Guangdong |
| ang_X14RP03 | + | - | + | - | - | + | - | - | + | - | - | 0.36 | Guangdong |
| ang_X14RP14 | - | - | - | + | - | - | - | - | + | - | - | 0.18 | Guangdong |
| ang_X14RP15 | - | - | + | + | - | - | + | - | + | - | - | 0.36 | Guangdong |
| ang_X14RP22 | - | - | - | - | - | - | - | - | + | - | - | 0.09 | Guangdong |
| cam_X11QS11 | + | - | - | + | + | + | + | - | - | + | - | 0.55 | Guangdong |
| cam_X13RP03 | + | + | + | - | + | - | + | - | + | - | - | 0.55 | Guangdong |
| cam_X13XC02 | + | + | + | - | + | - | + | - | + | - | - | 0.55 | Hainan |
| cam_X13XC21 | + | + | + | + | + | - | - | - | + | - | - | 0.55 | Hainan |
| par_G14SZ01 | - | - | - | - | - | + | + | + | - | - | - | 0.27 | Guangdong |
| par_G14SZ02 | - | - | - | - | + | - | + | - | + | - | + | 0.36 | Guangdong |
| par_X12XC14 | + | + | - | - | + | + | - | - | + | - | - | 0.45 | Hainan |
| com_X13YD06 | + | + | + | + | + | - | - | - | + | - | - | 0.55 | Hainan |
| com_X13YD0701 | + | + | + | + | + | - | - | - | + | + | - | 0.64 | Hainan |

Notes: +: with the gene, -: without the gene.

**Table S2.** The antimicrobial resistance pattern of *Vibrio* strains

| Strain name | VAN | AMO | MID | ERY | FUR | TOB | GEN | RIF | T/S | TET | DOX | CHL | FLO | NOR | CIP | MARIs | Provience |
| --- | --- | --- | --- | --- | --- | --- | --- | --- | --- | --- | --- | --- | --- | --- | --- | --- | --- |
| har_11QS22 | R | R | R | S | I | R | I | I | S | I | S | S | S | S | I | 0.27 | Guangdong |
| har_11WS31 | R | S | R | I | S | R | I | R | S | S | I | S | S | S | S | 0.27 | Guangdong |
| har_11WS38ABC | R | R | R | I | S | R | I | S | S | R | I | S | S | S | I | 0.33 | Guangdong |
| har_14K | R | R | R | I | R | I | I | I | S | S | S | S | S | S | S | 0.27 | NK |
| har_BSE1 | R | R | R | S | R | I | I | I | S | S | S | R | S | S | S | 0.33 | NK |
| har_BSE4 | R | R | I | S | S | S | S | S | S | S | S | S | S | S | S | 0.13 | NK |
| har_D79 | R | R | R | I | R | R | S | R | S | S | S | S | S | S | I | 0.40 | NK |
| har_F14MM01 | R | R | R | S | R | I | I | S | S | S | S | S | S | S | S | 0.27 | NK |
| har_F14MM02 | R | R | I | S | I | I | S | S | S | S | S | S | S | S | S | 0.13 | NK |
| har_FE1 | R | R | R | R | R | I | S | I | S | S | S | S | S | S | S | 0.33 | NK |
| har_FE2 | R | R | R | I | R | S | S | I | S | S | S | S | S | S | I | 0.27 | NK |
| har_JT1 | R | R | R | S | R | R | S | R | S | S | S | S | S | S | S | 0.40 | NK |
| har_JT2 | R | R | S | S | R | I | I | S | S | S | S | S | S | S | I | 0.20 | NK |
| har_JT3 | R | R | R | S | R | R | I | S | S | I | S | S | S | S | S | 0.33 | NK |
| har_V12RP10 | R | R | R | I | R | I | I | I | S | S | S | S | S | S | S | 0.27 | Guangdong |
| har_V12XC32 | R | R | R | I | R | I | S | I | S | S | S | S | S | S | S | 0.27 | Hainan |
| har_V13ZH01 | R | R | R | R | R | S | S | R | S | R | R | S | S | S | S | 0.53 | Guangdong |
| har_X11HK26 | R | R | R | S | I | I | S | S | S | S | S | S | S | S | S | 0.20 | Guangdong |
| har_X12RP11 | R | R | R | I | R | I | I | S | S | S | S | S | S | S | S | 0.27 | Guangdong |
| har_X12XW01 | R | S | S | S | R | R | S | S | S | S | S | S | S | S | S | 0.20 | Guangdong |
| har_X13DS03 | R | R | R | I | R | R | R | S | S | I | S | S | S | S | S | 0.40 | Fujian |
| har_X13RP16 | R | S | R | I | R | I | R | R | S | I | S | S | S | S | I | 0.33 | Guangdong |
| har_X13SY09 | R | S | S | S | R | I | I | S | S | S | S | S | S | S | S | 0.13 | Hainan |
| har_X13ZH05 | R | I | I | I | R | I | I | S | S | S | S | S | S | S | S | 0.13 | Guangdong |
| har_X14XC01 | R | I | S | I | R | I | I | I | S | S | S | S | S | S | S | 0.13 | Hainan |
| har_X14XC17 | R | S | S | I | R | I | R | I | S | S | S | S | S | S | S | 0.20 | Hainan |
| har_X15SZ09 | R | R | R | I | R | I | S | I | S | S | S | S | S | S | S | 0.27 | Guangdong |
| vul_X13ZH02 | R | R | R | R | S | R | S | I | S | S | S | S | S | S | S | 0.33 | Guangdong |
| vul_X13ZH04 | R | S | S | I | I | I | S | S | S | S | S | S | S | S | S | 0.07 | Guangdong |
| vul_X13ZJ03 | S | S | S | I | R | S | S | S | S | S | S | S | S | S | S | 0.07 | Guangdong |
| vul_X14DS02 | R | R | R | I | I | R | I | R | S | S | S | R | S | S | S | 0.40 | Fujian |
| vul_X14HD02 | R | R | S | S | I | R | S | S | S | S | S | S | S | S | S | 0.20 | Guangdong |
| vul_X14RP3101 | R | R | R | I | S | R | S | R | S | S | S | S | S | S | S | 0.33 | Guangdong |
| vul_X14SZ02 | R | S | S | S | S | R | S | R | R | R | S | S | S | S | S | 0.33 | Guangdong |
| vul_X14SZ04 | R | I | I | S | S | R | S | R | R | R | I | S | S | S | S | 0.33 | Guangdong |
| vul_X14SZ13 | R | R | S | S | S | R | S | S | S | S | S | S | S | S | S | 0.20 | Guangdong |
| vul_X14XC21 | R | S | S | S | S | I | S | S | S | S | S | S | S | S | S | 0.07 | Hainan |
| vul_X16XC06 | R | S | S | I | S | I | I | S | S | S | S | S | S | S | S | 0.07 | Hainan |
| alg_11QS15 | R | I | R | I | I | R | I | R | S | S | S | S | S | S | S | 0.27 | Guangdong |
| alg_E09811 | R | R | R | I | R | I | I | R | S | S | S | S | S | S | I | 0.33 | NK |
| alg_E09823 | R | R | I | I | S | I | S | S | S | S | S | S | S | S | S | 0.13 | NK |
| alg_F1 | R | R | I | I | R | I | S | R | S | S | S | S | S | S | I | 0.27 | NK |
| alg_F14DM01 | R | R | I | R | R | I | R | R | R | R | I | R | S | S | S | 0.60 | NK |
| alg_L12LF0301 | R | R | R | I | R | I | S | S | S | S | S | S | S | S | I | 0.27 | NK |
| alg_L12LF0302 | R | R | R | I | R | I | S | I | S | S | S | S | S | S | I | 0.27 | NK |
| alg_L12LF07 | R | R | R | I | R | I | S | R | S | S | S | S | S | S | I | 0.33 | NK |
| alg_N14FS02 | R | R | R | I | R | I | I | R | S | I | I | S | S | S | I | 0.33 | NK |
| alg_V12YD1601 | R | R | R | S | S | I | S | I | S | S | S | S | S | S | S | 0.20 | Hainan |
| rot_JT1 | R | R | I | S | R | I | S | S | S | S | S | S | S | S | I | 0.20 | NK |
| rot_X12XC39 | R | R | R | R | R | R | R | I | S | S | S | S | S | S | S | 0.47 | Hainan |
| rot_X13SY03 | R | R | R | I | R | R | I | R | S | S | I | S | S | S | I | 0.40 | Hainan |
| rot_ZHG1 | R | R | R | I | R | I | I | R | S | S | S | S | S | S | S | 0.33 | NK |
| rot_ZHG2 | R | R | R | I | R | R | I | R | S | I | I | I | I | S | S | 0.40 | NK |
| sco_X14RP05 | R | S | R | I | R | S | S | S | S | S | S | S | S | S | S | 0.20 | Guangdong |
| sco_X14RP08 | S | S | R | I | S | I | S | I | S | S | S | S | S | S | S | 0.07 | Guangdong |
| sco_X14RP0801 | R | I | R | R | S | R | R | I | S | I | S | S | S | S | S | 0.33 | Guangdong |
| sco_X14RP09 | R | R | R | I | I | I | S | R | R | R | R | S | S | S | S | 0.47 | Guangdong |
| ang_X14RP03 | R | I | R | S | R | S | S | S | S | S | S | S | S | S | S | 0.20 | Guangdong |
| ang_X14RP14 | R | R | R | I | S | I | R | I | S | S | S | S | S | S | S | 0.27 | Guangdong |
| ang_X14RP15 | I | I | I | S | S | I | S | S | S | S | S | S | S | S | S | 0.00 | Guangdong |
| ang_X14RP22 | R | I | I | S | S | I | S | I | S | S | S | S | S | S | S | 0.07 | Guangdong |
| cam_X11QS11 | R | R | R | I | S | I | S | S | S | S | S | S | S | S | S | 0.20 | Guangdong |
| cam_X13RP03 | R | R | R | I | I | R | I | R | S | I | S | S | S | S | S | 0.33 | Guangdong |
| cam_X13XC02 | R | R | R | I | I | R | I | R | S | I | S | S | S | S | I | 0.33 | Hainan |
| cam_X13XC21 | R | R | R | I | I | R | R | I | S | I | S | S | S | S | S | 0.33 | Hainan |
| par_G14SZ01 | R | R | R | I | R | I | S | R | S | S | S | S | S | S | S | 0.33 | Guangdong |
| par_G14SZ02 | R | I | R | I | I | I | S | S | S | S | S | S | S | S | S | 0.13 | Guangdong |
| par_X12XC14 | R | R | R | I | R | I | S | R | S | I | I | S | S | S | I | 0.33 | Hainan |
| com_X13YD06 | R | I | I | I | R | R | R | I | S | S | S | S | S | S | S | 0.27 | Hainan |
| com_X13YD0701 | R | R | R | I | I | R | I | R | R | R | R | S | S | S | S | 0.53 | Hainan |

Notes: I: Intermediate, R: Resistant, S: Sensitive, NK: not known.

**Table S3**. Comparison of antibiotics concentrations in the water between Guangdong province and Hainan province, China

| Antibiotics | Concentration (ug/L) | |
| --- | --- | --- |
|  | Guangdong | Hainan |
| Sulfadiazine | 3.145 | 9.763 |
| Sulfadimethylpyrimidine | 15.700 | - |
| Sulfamethoxazole | 8.545 | - |
| Norfloxacin | 68.520 | - |
| Ofloxacin | 6.930 | - |
| Tetracycline | 6.030 | 9.250 |
| Anhydroerythromycin | 21.705 | - |
| Enrofloxacin | 4.700 | - |
| Roxithromycin | 2.725 | - |
| Ciprofloxacin | 8.029 | - |
| Erythromycin | 529.5 | - |
| Trimethoprim | - | - |
| Clarithromycin | - | - |
| Chloramphenicol | - | - |
| **References** | 1-3 | 4 |

Note: -, not analyzed; n.d., not detected; all the concentrations in Guangdong have been shown as the average concentrations, all the concentrations in Hainan have been shown as the detected concentrations range.

**Table S4.** The primer pair sequences used for this study

| Primer name | sequence (5'-3') | Tm (°C) | product size | Reference |
| --- | --- | --- | --- | --- |
| 8F | AGAGTTTGATCCTGGCTCAG | 53 | 1500 | universal |
| 1492R | GGTTACCTTGTTACGACTT | 53 |  |  |
| *rctB*-F | ATGCCNGARGGYTTYAARAG | 55 | 544 | 5 |
| *rctB*-R | GTWGGMGCCATRTTRCGYTT | 55 |  |  |
| *aphA*-F | ACCACACGTAATTCTAACTG | 53 | 515 | 6 |
| *aphA*-R | GTTCTGTTAGTACTTCTTCTGC | 53 |  |  |
| *vhh*-F | GATTGGGAATGGGCAGAAAA | 55 | 319 | 6 |
| *vhh*-R | GGAATCGCCATTGTGATGC | 55 |  |  |
| *hflK*-F | TGCACGACCAGTTGCTTTAG | 57 | 232 | 7 |
| *hflK*-R | AAGTGGTCGTCAGCAAATCC | 57 |  |  |
| *luxR*-F | GTGGTTCGTCAATTCTCGAAC | 55 | 178 | 6 |
| *luxR*-R | CGAATAGTGGCCACACTTC | 55 |  |  |
| *chiA*-F | CTCAAGGTGTTTGGGAAGATG | 55 | 83 | 6 |
| *chiA*-R | GTTGATGCCAGTGTTGTTCG | 55 |  |  |
| *toxR_Vh_*-F | GAAGCAGCACTCACCGAT | 55 | 382 | 8 |
| *toxR_Vh_*-R | GGTGAAGACTCATCAGCA | 55 |  |  |
| *flaC*-F | AAATCATTCCAAATCGGTGC | 53 | 580 | 9 |
| *flaC*-R | TCTTTGATTCGGCTCTTA | 53 |  |  |
| *tdh*-F | CCACTACCACTCTCATATGC | 55 | 250 | 10 |
| *tdh*-R | ATACGAGTGGTTGCTGTCATG | 55 |  |  |
| *vvh*-F | GCTATTTCACCGCCGCTCAC | 60 | 222 | 11 |
| *vvh*-R | CCGCAGAGCCGTAAACCGAA | 60 |  |  |
| *toxR_Vc_ -F* | ATGTTCGGATTAGGACAC | 53 | 883 | 12 |
| *toxR_Vc_ -R* | TACTCACACACTTTGATGGC | 53 |  |  |
| *hlyA-F* | GGCAAACAGCGAAACAAATACC | 58 | 738 | 13 |
| *hlyA-R* | CTCAGCGGGCTAATACGGTTTA | 58 |  |  |


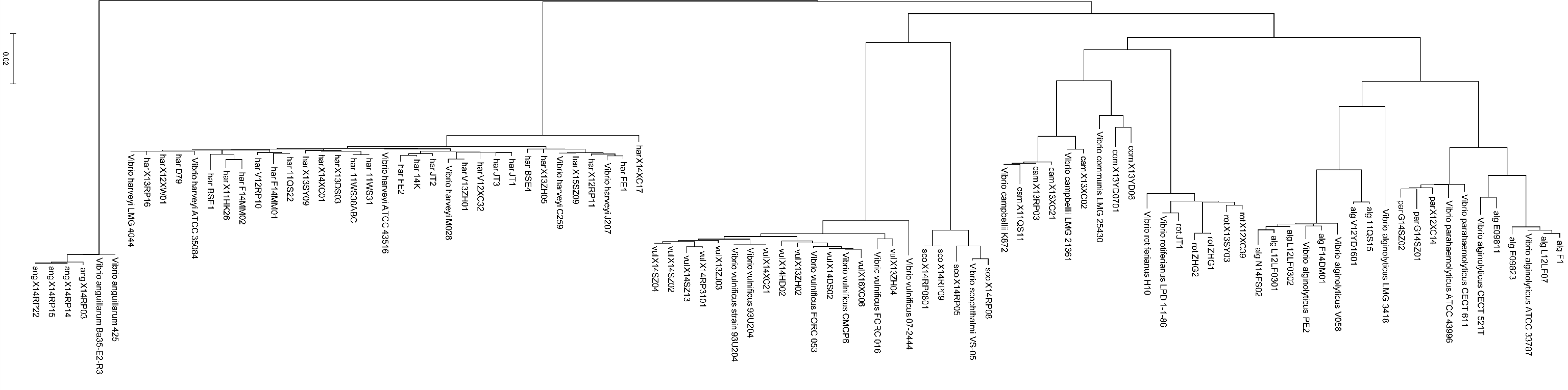


**Figure.** **S1** Phylogenetic tree constructed based on *rctB* sequences of the *Vibrio* iaolates in this study and the reference strains. The scale bar indicates 0.02 substitutions per sequence position. har: *V. harveyi*, vul: *V. vulnificus*, alg: *V. alginolyticus*, rot: *V. rotiferianus*, sco: *V. scophthalmi*, ang: *V. anguillarum*, cam: *V. campbellii*, par: *V. parahaemolyticus*, com: *V. communis*.


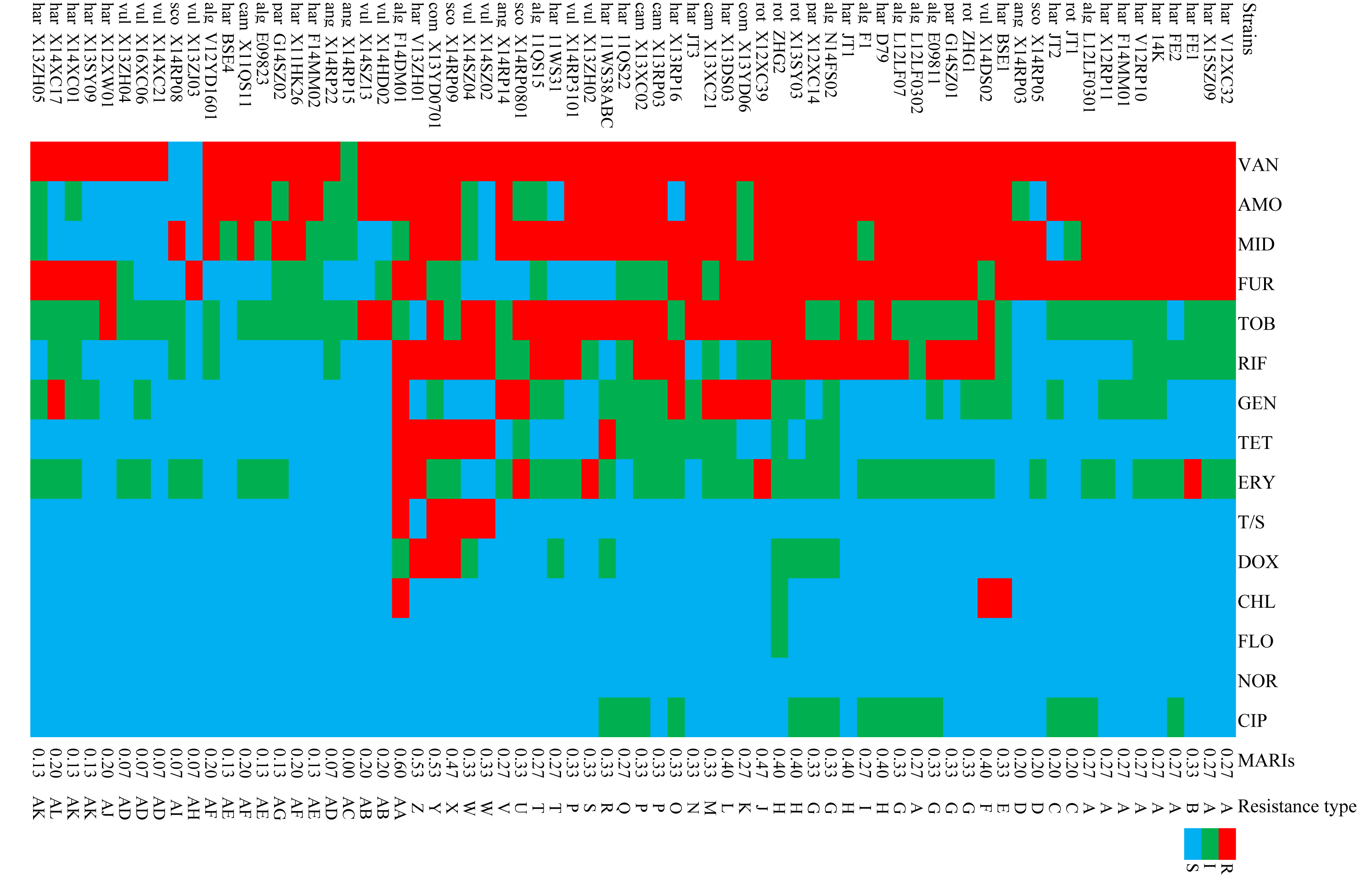


**Figure. S2** The antimicrobial resistance profiles was showed with a heat map. I: Intermediate, R: Resistant, S: Sensitive.

**
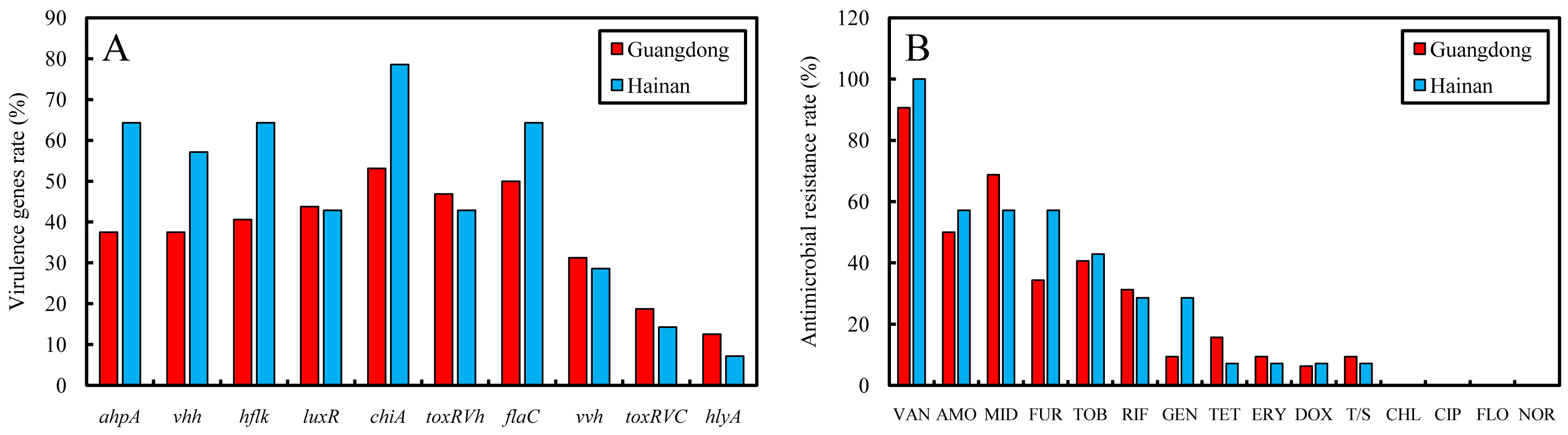
**

**Figure. S3** The virulence genes rate (A) and antimicrobial resistance rate (B) in Guangdong and Hainan.


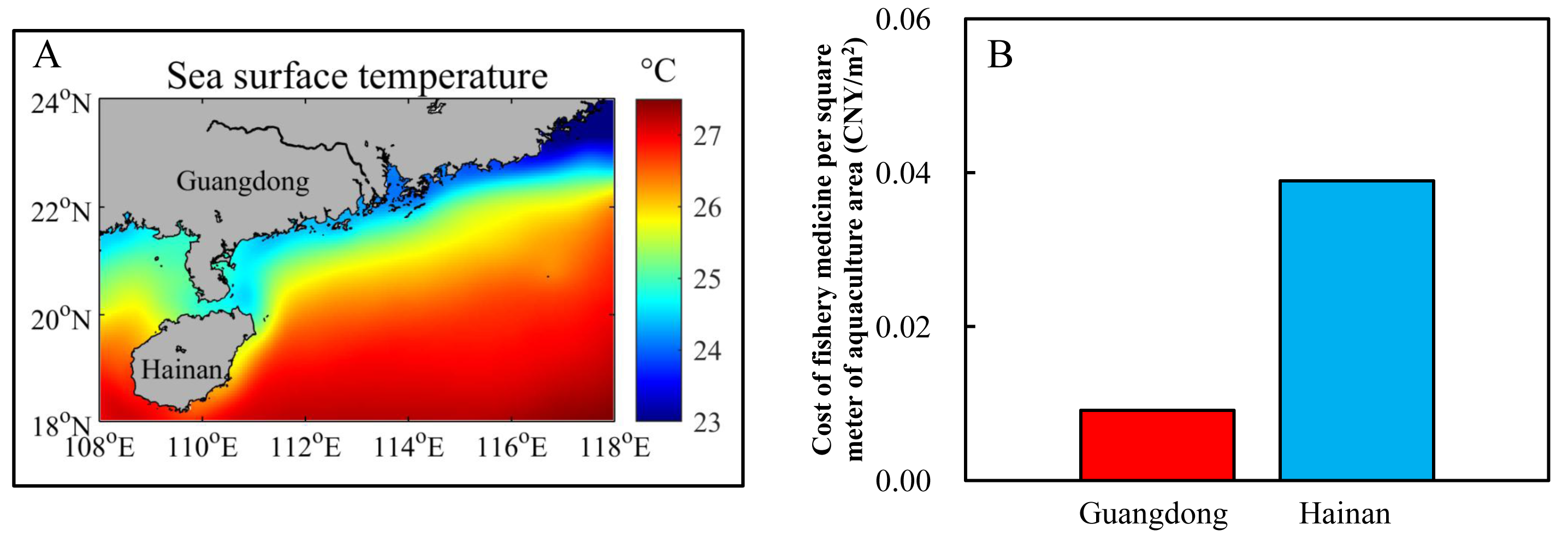


**Figure. S4** The average sea surface temperature (SST) (A) and cost of fishery medicine per square meter of aquaculture area (B) during sampling period in Guangdong and Hainan. The SST data were the level-4 products of the Group for High Resolution Sea Surface Temperature (GHRSST, https://www.ghrsst.org). The data analysis method was refered to Gao et al.^14^. The data of cost of fishery medicine and aquaculture areas were from China fishery statistical yearbook^15-18^.

**References**

1. Liang, X. *et al*. The distribution and partitioning of common antibiotics in water and sediment of the Pearl River Estuary, South China. *Chemosphere*. **92,** 1410-1416 (2013).
2. Xu, W. H. *et al*. Antibiotics in riverine runoff of the Pearl River Delta and Pearl River Estuary, China: Concentrations, mass loading and ecological risks. *Environ. Pollut*. **182,** 402-407 (2013).
3. Xu, W. H. *et al*. Determination of selected antibiotics in the Victoria Harbour and the Pearl River, South China using high-performance liquid chromatography-electrospray ionization tandem mass spectrometry. *Environ. Pollut*. **145,** 672-679 (2007).
4. Han, J. L., He, D. C., Wang, Z. L., He, Y. P., & Xu, Z. C. Research of variety and residual characteristics of antibiotics of wastewater in industrial raising farm. *Guangzhou. Chemistry*. **37,** 27-31 (2012). (in Chinese)
5. Zeng, Q. D. Multilocus sequence analysis and antibiogram type research of *Vibrio harveyi* strains isolated from maricultured fishes. *Shanghai. Ocean. University*. (2014). (in Chinese)
6. Deng, Y. Q. *et al*. Primer group and kit used for detecting *Vibrio harveyi* multiple virulence genes and application thereof. China 201711158684.8, (2017).
7. Ruwandeepika, H. A., *et al*. Presence of typical and atypical virulence genes in vibrio isolates belonging to the Harveyi clade. *J. Appl. Microbiol*. **109,** 888-899 (2010).
8. Pang, L. *et al*. Identification of *Vibrio harveyi* using PCR amplification of the *toxR* gene. *Lett. Appl. Microbiol*. **43,** 249-255 (2006).
9. Bai, F. *et al*. Distribution of five vibrio virulence-related genes among *Vibrio harveyi* isolates. *J. Gen. Appl. Microbiol*. **54,** 71-78 (2008).
10. Tada, J. *et al*. Detection of the thermostable direct hemolysin gene (*tdh*) and the thermostable direct hemolysin-related hemolysin gene (*trh*) of *Vibrio parahaemolyticus* by polymerase chain reaction. *Mol. Cell Probe*. **6,** 477-487 (1992).
11. Lee, J., Bang, Y., Rhee, J., & Choi, S. Two-stage nested PCR effectiveness for direct detection of *Vibrio vulnificus* in natural samples. *J. Food Sci*. **64,** 158-162 (1999).
12. Miller, V. L., Taylor, R. K., & Mekalanos, J. J. Cholera toxin transcriptional activator ToxR is a transmembrane DNA binding protein. *Cell*. **48,** 271-279 (1987).
13. Saravanan, V., Kumar, H. S., Karunasagar, I., & Karunasagar, I. Putative virulence genes of *Vibrio cholerae* from seafoods and the coastal environment of Southwest India. *Int. J. Food Microbiol*. **119,** 329-333 (2007).
14. Gao, N. *et al*. Quantile Analysis of Long-Term Trends of NearSurface Chlorophyll-a in the Pearl River Plume. *Water*. 12, 1662; 10.3390/w12061662 (2020).
15. Fisheries Bureau of the Ministry of Agriculture. *China Fishery Statistical Yearbook*. (ed. Fisheries Bureau of the Ministry of Agriculture) 3-58 (China Agricultural Press, 2012). (in Chinese)
16. Fisheries Bureau of the Ministry of Agriculture. *China Fishery Statistical Yearbook*. (ed. Fisheries Bureau of the Ministry of Agriculture) 3-58 (China Agricultural Press, 2013). (in Chinese)
17. Fisheries and Fisheries Administration Bureau of the Ministry of Agriculture. *China Fishery Statistical Yearbook*. (ed. Fisheries and Fisheries Administration Bureau of the Ministry of Agriculture) 3-58 (China Agricultural Press, 2014). (in Chinese)
18. Fisheries and Fisheries Administration Bureau of the Ministry of Agriculture. *China Fishery Statistical Yearbook*. (ed. Fisheries and Fisheries Administration Bureau of the Ministry of Agriculture) 3-58 (China Agricultural Press, 2015). (in Chinese)
